# Evolving Paradigms in Gastric Cancer Staging: From Conventional Imaging to Advanced MRI and Artificial Intelligence

**DOI:** 10.3390/diagnostics16020284

**Published:** 2026-01-16

**Authors:** Giovanni Balestrucci, Vittorio Patanè, Nicoletta Giordano, Anna Russo, Fabrizio Urraro, Valerio Nardone, Salvatore Cappabianca, Alfonso Reginelli

**Affiliations:** 1 Radiology Unit, Pineta Grande Hospital, 81030 Castel Volturno, Italy; 2 Departiment of Medicine and Health Sciences “Vincenzo Tiberio”, Università degli Studi del Molise, 86100 Campobasso, Italy; 3 Department of Precision Medicine, University of Campania “L. Vanvitelli”, 80138 Naples, Italynicoletta.giordano@unicampania.it (N.G.); salvatore.cappabianca@unicampania.it (S.C.); alfonso.reginelli@unicampania.it (A.R.); 4 Department of Life Sciences, Health and Health Professions, Link Campus University, 00165 Rome, Italy; fabrizio.urraro@unicampania.it

**Keywords:** gastric cancer, neoplasm staging, magnetic resonance imaging, diffusion-weighted imaging, artificial intelligence, radiomics

## Abstract

**Background**: Accurate preoperative staging is the cornerstone of therapeutic decision-making in gastric cancer (GC), yet standard modalities often fail to capture the full extent of disease, particularly in diffuse and poorly cohesive histotypes. This review aims to provide a comprehensive update on diagnostic imaging for GC, evaluating the established roles of CT, EUS, and PET/CT alongside the emerging capabilities of Magnetic Resonance Imaging (MRI) and Artificial Intelligence (AI). **Methods**: A structured narrative review was conducted by searching indexed biomedical databases for studies published between 2015 and 2024. A structured literature search screening process identified 410 relevant studies focusing on T, N, and M staging accuracy, quantitative imaging biomarkers, and radiomics. **Results**: While Multidetector CT remains the universal first-line modality, its sensitivity declines in infiltrative tumors and low-volume peritoneal carcinomatosis. EUS retains superiority for early (T1-T2) lesions but may offer limited value in advanced stages. Conversely, MRI (leveraging diffusion-weighted imaging (DWI) and multiparametric protocols) indicates superior soft-tissue contrast, potentially outperforming CT in the assessment of serosal invasion, nodal involvement, and occult peritoneal metastases. Furthermore, emerging fibroblast activation protein inhibitor (FAPI) PET tracers show promise in overcoming the limitations of FDG in mucinous and diffuse GC. Finally, radiomics and deep learning models are providing novel quantitative biomarkers for non-invasive risk stratification. **Conclusions**: Contemporary GC staging requires a tailored, multimodality approach. Evidence supports the increasing integration of MRI and quantitative imaging into clinical workflows to overcome the limitations of conventional techniques and support precision oncology.

## 1. Introduction

Gastric cancer (GC) is one of the most common malignancies worldwide, remaining a major cause of cancer-related mortality despite a progressive decline in incidence in many Western countries [1,2,3,4]. In the United States, approximately 22,000 new cases are diagnosed every year [2], while in Italy, the estimated annual incidence is around 8400 new cases in men and 6100 in women [5]. These figures are considered more favourable than in previous decades, largely due to better control of major risk factors, including *Helicobacter pylori* infection, dietary patterns, and cigarette smoking [6]. However, GC still carries a poor prognosis in many settings, with 5-year survival frequently below 40%, particularly in advanced stages and in diffuse or poorly cohesive histotypes [7].

From a pathological standpoint, GC can be classified according to macroscopic appearance using Borrmann’s classification, which stratifies advanced gastric cancers into four main types [1], and according to histology using Lauren’s classification (intestinal, diffuse, and mixed types) and the more recent WHO classification [8]. Prognosis is primarily driven by tumour stage as defined by the AJCC/UICC TNM system, which reflects the depth of wall invasion (T), the extent of lymph node involvement (N), and the presence of distant metastases (M) [9]. Nonetheless, several additional prognostic determinants have been identified, including histologic subtype, grade, molecular profile, response to neoadjuvant chemotherapy, and the pattern of metastatic spread [10].

Lauren’s classification remains of relevance from both a biological and an imaging perspective. The intestinal type typically affects older patients and is more common in men; it is often associated with chronic inflammatory conditions such as long-standing *H. pylori* infection, atrophic gastritis, and intestinal metaplasia, as well as gastroesophageal reflux disease at the cardia [11]. In contrast, the diffuse type is more frequently observed in younger patients and in women; it is strongly associated with alterations in cell adhesion (e.g., E-cadherin dysfunction) and is less clearly linked to inflammatory precursors [12]. Diffuse and poorly cohesive carcinomas tend to infiltrate the gastric wall in a non-cohesive fashion, with early submucosal and serosal extension, a propensity for peritoneal dissemination, and a higher risk of peritoneal carcinomatosis and ovarian metastases (Krukenberg tumours), resulting in worse overall survival compared with intestinal-type cancers [13]. Several meta-analyses have confirmed the adverse prognostic impact of diffuse histology, independent of stage and treatment [14].

Accurate staging is a cornerstone of GC management because it directly influences therapeutic decision-making and long-term outcome. Early lesions confined to mucosa or superficial submucosa may be amenable to endoscopic resection, whereas most patients with locally advanced disease are candidates for multimodal treatment, including neoadjuvant or perioperative chemotherapy and radical surgery with D2 lymphadenectomy [15]. Inadequate local or nodal staging may lead to under-treatment of aggressive disease or unnecessary intensification of therapy, with a negative impact on both survival and quality of life [16]. Thus, precise assessment of T, N, and M status, as well as of key biological features, is essential in a modern, multidisciplinary approach to GC.

In current clinical practice, the main imaging modalities used for GC staging are contrast-enhanced multidetector computed tomography (CT), endoscopic ultrasound (EUS), and 18F-fluorodeoxyglucose positron emission tomography combined with CT (FDG PET/CT) [17]. CT is widely available and represents the standard first-line technique for preoperative staging, allowing assessment of the primary tumor, regional lymph nodes, and distant metastases, particularly in the liver, lungs, and peritoneum [18]. EUS provides high-resolution, layer-by-layer visualization of the gastric wall and is considered one of the reference techniques for local T staging in many guidelines, although its performance is heavily operator-dependent and limited in stenotic or extensive lesions [10]. FDG PET/CT adds functional information on tumor metabolism and can be helpful for detecting distant metastases and for assessing treatment response, but its sensitivity is influenced by tumor size, histologic subtype, and background gastric uptake; diffuse or signet-ring cell carcinomas often show low or absent FDG avidity, leading to false-negative results [19].

Historically, magnetic resonance imaging (MRI) has played a minor role in GC staging because of perceived limitations such as lower spatial resolution, longer acquisition times, and sensitivity to motion artefacts due to respiration and peristalsis [20]. In recent years, however, substantial technical advances have improved both acquisition speed and robustness, including high-field scanners, fast sequences, respiratory triggering, and the use of antiperistaltic agents. MRI offers excellent soft-tissue contrast, multiparametric capability, and the absence of ionizing radiation, which is particularly relevant in younger patients and in those requiring repeated imaging [15]. Beyond conventional T1- and T2-weighted sequences, diffusion-weighted imaging (DWI) with apparent diffusion coefficient (ADC) maps, intravoxel incoherent motion (IVIM) analysis, and dynamic contrast-enhanced MRI (DCE-MRI) allow for the non-invasive characterization of tissue cellularity, microcirculation, and vascular permeability. Emerging data suggest that these functional parameters may correlate with histologic subtype, aggressiveness, and treatment response, and could be particularly useful in challenging histotypes such as diffuse and poorly cohesive carcinomas [21,22,23].

Parallel to these imaging advances, quantitative image analysis and artificial intelligence (AI) are gaining increasing attention in GC. Radiomics enables the extraction of high-dimensional quantitative features from CT, MRI, and PET images that capture intra-tumour heterogeneity beyond visual assessment, while AI-based models can integrate imaging, clinical, and pathological data to support diagnosis, staging, prognostic stratification, and prediction of treatment response [24,25]. Although still largely in the research phase and limited by heterogeneity of methods and small, often monocentric cohorts, these approaches hold promise for refining risk assessment and tailoring therapy in GC.

Despite these established protocols, the persistent rate of under-staging (particularly in diffuse-type cancers) highlights a critical diagnostic gap that current guidelines fail to address fully. Consequently, the present structured narrative review aims to provide an updated overview of imaging in GC staging, focusing on the established roles and limitations of CT, PET/CT, and EUS, and on the emerging contribution of MRI and advanced techniques, including DWI, IVIM, DCE-MRI, radiomics, and AI. Particular attention is given to histological and biological factors that influence imaging performance, with the goal of clarifying how these modalities can be integrated to improve staging accuracy and support more personalized management of gastric cancer.

## 2. Literature Search Strategy and Selection

All retrieved citations were exported and combined into a single dataset for screening. A total of 1409 records were initially identified.

### 2.1. Study Selection

A comprehensive literature search was performed to identify studies evaluating the diagnostic performance of imaging modalities for preoperative staging of gastric cancer, with a specific focus on emerging techniques, including advanced magnetic resonance imaging (MRI) protocols, PET/CT (FDG-PET and FAPI-PET), and quantitative imaging/artificial intelligence (AI) applications. Five search strings were constructed using standardized Boolean operators and terminology. The queries covered four major imaging domains (CT, EUS, PET/FAPI, and MRI) and one mixed-modality query. Searches were conducted across indexed biomedical databases up to December 2024, without language or date restrictions. The final literature search was performed in February 2025. Databases consulted included PubMed (MEDLINE), Scopus, and Web of Science.

The five search strings used across the databases were:

(1) [gastric cancer AND (“CT” OR “computed tomography”) AND (“staging” OR “diagnostic accuracy”)].
(2) [gastric cancer AND (“EUS” OR “endoscopic ultrasound”) AND (“T staging”)].
(3) [gastric cancer AND (“PET” OR “FDG PET/CT” OR “FAPI”) AND (“lymph node” OR “metastasis”)].
(4) [gastric cancer AND (“MRI” OR “DWI” OR “IVIM” OR “DCE-MRI”) AND (“staging” OR “peritoneal metastasis”)].
(5) [gastric cancer AND (“radiomics” OR “artificial intelligence” OR “machine learning”) AND (“staging” OR “prediction”)].

All retrieved citations were exported and combined into a single dataset for screening. A total of 1409 records were initially identified.

A structured literature search strategy was employed to ensure comprehensive coverage, although the synthesis remains narrative to accommodate the heterogeneity of the included imaging technologies.

Titles, abstracts, and NCBI-generated summaries (when available) were screened to determine eligibility based on the predefined inclusion and exclusion criteria. Full-text reading was performed for all articles retained for the final synthesis, but initial exclusion was based on abstract evaluation to capture the state of the art rather than to conduct a quantitative meta-analysis.

Duplicate references were removed before screening, resulting in 189 duplicates excluded, leaving 1220 unique records.

### 2.2. Inclusion Criteria

Eligibility was limited to primary research studies (including prospective or retrospective cohorts, multicenter trials, and original radiomics or machine-learning investigations) that specifically evaluated the diagnostic accuracy, staging performance, or prognostic value of CT, EUS, MRI, or PET/FAPI in gastric cancer. Particular emphasis was placed on studies directly addressing T and N staging, the assessment of serosal invasion, and the detection of occult metastases.

### 2.3. Exclusion Criteria

Conversely, records were excluded if they were not primarily focused on gastric cancer imaging, consisted of isolated case reports lacking methodological relevance for staging, or dealt exclusively with treatment response without providing baseline staging performance data. Retracted articles, duplicates, and non-scientific materials such as editorials or letters were also removed.

During the initial screening phase, 803 studies were excluded primarily due to a lack of direct relevance to the imaging modalities or staging outcomes of focus (e.g., studies focused solely on therapy without staging data, or on non-gastric cancers), or because they did not meet the predefined inclusion criteria.

### 2.4. Final Dataset

A total of 410 studies were retained and included in qualitative synthesis. These encompass:

(1) CT-based staging and radiomics (including Δ-radiomics and lymph-node prediction models);
(2) EUS performance for T staging and early gastric cancer stratification;
(3) PET/CT and PET/MRI (FDG and FAPI) for nodal and metastatic assessment;
(4) MRI techniques, including DWI, perfusion imaging, and comparative accuracy vs. CT and EUS;
(5) Hybrid or multimodality approaches, including radiomics-based fusion models;
(6) High-quality meta-analyses relevant to each imaging domain.

**Figure 1:**
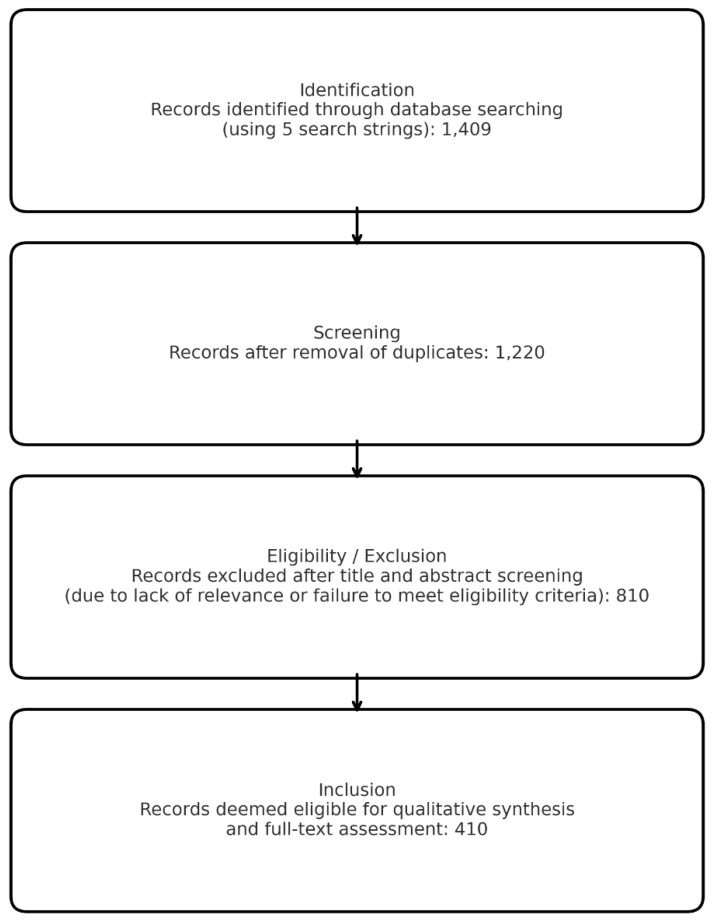
Flow diagram summarizing the literature search and study selection process. Overall, 1409 records were identified through database searches using five search strings. After removal of duplicates, 1220 records underwent title and abstract screening, leading to the exclusion of 810 records for lack of relevance or failure to meet the predefined inclusion/exclusion criteria. The remaining 410 records were considered eligible for qualitative synthesis and full-text assessment.

A flow diagram summarizing the selection process is provided separately (Figure 1).

To manage and synthesize the high volume of retrieved literature (*n* = 410), an artificial intelligence tool was employed to assist in thematic clustering and initial drafting of the results. The authors provided the selected bibliographic dataset and established the clinical framework for the synthesis. All AI-assisted outputs were rigorously reviewed, critiqued, and edited by the authors to ensure clinical accuracy, consistency with the source data, and adherence to radiological standards.

## 3. Results

### 3.1. Overview of Included Studies

The integrated literature search across four complementary query strings yielded a broad and heterogeneous body of evidence spanning CT, EUS, PET/CT, PET/MRI, WB-DWI/MRI, advanced multiparametric MRI, and emerging molecular imaging such as FAPI PET. After screening titles, abstracts, and full-texts when available, studies were grouped into thematic domains reflecting the diagnostic pillars of gastric cancer staging: T staging, N staging, M staging, and histotype-related diagnostic variability (Table 1).

Across modalities, the most consistent finding was the reduced diagnostic performance in diffuse-type and signet-ring carcinomas, regardless of the imaging technique. This histotype-related limitation recurred in CT, PET/CT, and EUS studies, whereas MRI-based approaches (especially diffusion- and perfusion-derived parameters) demonstrated more stable performance across different biological subtypes.

### 3.2. CT-Based Staging Performance

#### 3.2.1. T Staging with Contrast-Enhanced CT

Classical multidetector CT (MDCT) has shown highly variable results across studies. While some authors have reported accuracy values between 70–90% for T staging [38,39], others observed markedly lower performance, with figures ranging between 50–60%.

The variability in diagnostic performance reported across studies can be attributed to several technical and biological factors, including inconsistencies in gastric distension protocols, differences in slice thickness and reconstruction kernels, and significant inter-observer heterogeneity. Furthermore, a trend toward reduced sensitivity is frequently observed in scirrhous or diffuse-type tumors, where the lack of a defined mass may limit the discriminative power of standard CT.

Dual-energy CT appeared to marginally improve discriminative power, although it did not seem to fully overcome these intrinsic limitations.

#### 3.2.2. Limitations in Diffuse Histotypes

CT’s reduced sensitivity in diffuse gastric cancer was a recurrent observation. Diffuse tumors often present with subtle mural thickening, submucosal infiltration, and lower contrast enhancement gradients (features that can make it challenging for CT to accurately delineate the depth of invasion).

These findings suggest the potential benefit of using complementary techniques, such as EUS or MRI, in selected clinical scenarios.

### 3.3. EUS Performance in T Staging

Across multiple studies and meta-analyses, EUS is generally regarded as a highly sensitive technique for identifying early gastric cancer (EGC) and distinguishing T1 vs. T2 lesions. Numerous reports [40,41,42] have indicated high accuracy for T1 staging (often >80–90%), but noted a progressively reduced accuracy for deeper invasion, particularly in T3–T4 lesions.

However, the performance of EUS appears to be heavily influenced by operator expertise and tumor location (being notably less accurate near the cardia and pylorus) as well as by the presence of fibrosis after neoadjuvant therapy.

The learning-curve effect is well documented, with significant performance variability among less-experienced endosonographers.

### 3.4. EUS After Neoadjuvant Therapy

Accuracy has been reported to decrease post-chemotherapy, as reported by Redondo-Cerezo et al. [43], where inflammatory or fibrotic wall thickening could confound the assessment of true residual tumor.

Consequently, while EUS appears to remain highly useful for initial T staging, its clinical utility may be more limited in advanced tumors, diffuse-type cancers, and in the post-treatment evaluation setting.

### 3.5. PET/CT and PET/MRI Findings

#### 3.5.1. FDG PET/CT Sensitivity for T and N Staging

FDG PET/CT often demonstrates high specificity but may show limited sensitivity for primary lesion detection and lymph-node involvement, with reported sensitivities frequently ranging between 31–65% depending on histotype [44].

Performance appears particularly challenged in diffuse-type and signet-ring carcinomas due to low glucose transporter expression, as well as in mucinous tumors and small-volume nodal metastases.

#### 3.5.2. Detection of Distant Metastases (M Staging)

PET/CT achieved high specificity (near 100%) for metastases in several studies [45], yet sensitivity often remained limited, especially for peritoneal metastases and low-FDG-uptake lesions.

Because of these limitations, PET/CT may be insufficient to exclude peritoneal carcinomatosis, even in advanced disease.

#### 3.5.3. FAPI PET

Newer tracers such as ^68^Ga-FAPI have shown higher uptake in diffuse and scirrhous tumors, appearing to outperform FDG in the detection of nodal and peritoneal metastases [31]. This emerging modality shows promising potential to become a cornerstone tool for future staging algorithms.

#### 3.5.4. PET/MRI

Hybrid PET/MRI, particularly when incorporating respiratory-gated protocols, offers improved soft-tissue contrast and has demonstrated high diagnostic confidence for the assessment of peritoneal metastases, local-regional staging, and nodal characterization.

### 3.6. MRI and Diffusion-Weighted Imaging (DWI/IVIM)

MRI is increasingly emerging as a versatile modality, with studies frequently reporting higher accuracy than CT for T staging in selected cases [46,47,48,49]. Additionally, the modality has shown favorable performance for N staging (especially when utilizing ADC-based radiomics) and may provide better detection of peritoneal metastases, with several pilot studies reporting exceptionally high accuracy rates, particularly in whole-body DWI protocols [46,50,51].

#### 3.6.1. DWI, ADC, and IVIM Metrics

Multiple analyses suggest that quantitative metrics offer biological insights; specifically, lower ADC values appear to correlate with higher tumor aggressiveness, while diffusion coefficients have shown potential associations with Ki-67 or HER2 expression.

These markers show potential value as quantitative biomarkers for aggressiveness, response prediction, and risk stratification.

#### 3.6.2. DCE-MRI and Perfusion Analysis

Studies using DCE-MRI provided evidence that perfusion parameters differ significantly between intestinal and diffuse subtypes. Moreover, higher Ktrans values seem to correlate with more aggressive disease, and perfusion imaging has shown utility in differentiating fibrotic post-treatment changes from viable residual tumor.

#### 3.6.3. Whole-Body DWI (WB-DWI/MRI)

WB-DWI/MRI has shown potential for improved detection of peritoneal metastases and better characterization of nodal disease compared to conventional imaging, achieving high agreement rates with surgical findings.

Although limited by a small sample size, the findings consistently suggest a possible advantage for MRI over CT for M staging.

#### 3.6.4. Integrated Modality Evaluation and Multimodality Trends

Studies explicitly comparing CT, EUS, and MRI reached a consistent conclusion: while CT remains essential as the first-line staging modality and EUS retains superiority for early lesions, MRI surpasses CT in the evaluation of difficult histotypes and peritoneal disease. Concurrently, PET/CT shows limited value in diffuse types, whereas FAPI PET is emerging as a potential game-changer. Finally, radiomics and deep-learning approaches are providing meaningful quantitative biomarkers for T, N, and M staging (Table 2).

#### 3.6.5. Histotype-Driven Diagnostic Variability

Across nearly all imaging methods, diffuse-type cancers exhibited lower contrast enhancement, reduced metabolic uptake, and infiltrative patterns that limit layer discrimination. These morphological traits mechanistically explain why diffuse cancers are consistently understaged by CT, poorly detected by PET/CT, and difficult to characterize by EUS.

MRI, particularly DWI and DCE, demonstrated more consistent performance regardless of histotype, underscoring its potential in future staging guidelines.

#### 3.6.6. Summary of Key Evidence

The aggregated evidence points toward MRI and advanced PET tracers as highly promising modalities for improving staging accuracy, especially in biologically aggressive or low-FDG-uptake gastric cancers. While the traditional CT–EUS–PET workflow remains the cornerstone of clinical practice, substantial improvements in diagnostic accuracy appear achievable through the integration of MRI-based quantitative imaging and emerging radiotracers (e.g., FAPI) (Table 3).

## 4. Discussion

Gastric cancer (GC) remains a complex and heterogeneous disease, in which imaging serves as the cornerstone of diagnosis, staging, prognostic stratification, and treatment planning. The results of this comprehensive review highlight substantial advances in multimodality imaging, while reaffirming persistent challenges in specific biological and anatomical contexts. In particular, three domains emerge as crucial for contemporary staging: T staging accuracy, nodal characterization, and detection of peritoneal disease, all strongly influenced by tumor histotype.

### 4.1. CT as First-Line Modality: Essential but Insufficient in Challenging Scenarios

Multidetector CT (MDCT) remains the international standard for initial staging due to its availability, speed, and ability to assess locoregional extension and distant disease concurrently [79,80]. Across studies, CT demonstrated reasonable accuracy for T staging in many patients; however, performance varied widely and declined sharply in diffuse and poorly cohesive carcinomas [81].

These evidences suggest that CT alone may not always reliably discriminate the depth of invasion in all GC phenotypes [53,82,83,84].

The primary limitations of CT arise from its insufficient soft-tissue contrast [79], which hampers the delineation of the layered stomach wall. Furthermore, the modality exhibits reduced conspicuity for infiltrative or submucosal growth patterns [85] and indicates poor sensitivity for low-volume nodal and peritoneal metastases [86]. These challenges are often compounded by variability in gastric distention and patient preparation [87].

Dual-energy CT and virtual monochromatic reconstructions showed incremental benefits but did not overcome fundamental biological constraints [29]. Collectively, the evidence indicates that CT appears to perform most effectively in intestinal-type, exophytic, and mass-forming tumors, whereas its diagnostic confidence diminishes when evaluating infiltrative histotypes [88].

### 4.2. EUS Retains Superiority for Early T Staging but Is Limited in Advanced Disease

Endoscopic ultrasound (EUS) is widely recognized for providing unparalleled detail of the gastric wall layers, making it the most reliable modality for distinguishing T1 from T2 lesions [54]. This distinction is often considered clinically decisive, particularly when selecting candidates for endoscopic resection [89]. Multiple multicenter and single-institution studies reported sensitivities exceeding 80–90% for early gastric cancer, supporting EUS as a preferred modality in this context [90].

However, its limitations are equally well documented. The diagnostic yield decreases progressively with deeper invasion (T3–T4), in tumors located in the cardia or pylorus, and in those with extensive fibrosis or ulceration [91]. Post-neoadjuvant therapy assessment represents another critical challenge, with several authors demonstrating that treatment-induced inflammatory changes significantly impair accuracy [43]. Moreover, a pronounced operator dependence and a steep learning curve contribute to variability in reported performance [89].

These findings support a selective, rather than universal, application of EUS, ideally integrated with CT and MRI for a complete preoperative evaluation [55,92].

### 4.3. PET/CT and Emerging PET Tracers: High Specificity, Limited Sensitivity in Diffuse Histotypes

Fluorodeoxyglucose PET/CT (FDG PET/CT) provides valuable metabolic data, yet its utility in gastric cancer may be constrained by biological variability [93]. Diffuse-type and signet-ring cell carcinomas (which account for a significant proportion of GC globally) often exhibit low or absent FDG uptake due to reduced expression of glucose transporters and distinct stromal–tumour interactions [94]. Therefore, PET/CT often exhibits limited sensitivity for both primary tumor detection and nodal staging in these subtypes, with performance ranging from modest to poor across studies [95,96].

Despite these limitations, the modality retains exceptionally high specificity for distant metastases and can identify unexpected extraperitoneal disease, including rare metastatic sites, thereby influencing management in selected cases [30]. Its utility, therefore, lies primarily in confirming metastases when detected, rather than excluding them when absent, and it tends to be most effective when used in conjunction with contrast-enhanced CT and/or staging laparoscopy in locally advanced disease [97].

Recent advancements introduce more promising alternatives. Fibroblast activation protein inhibitor tracers (e.g., ^68^Ga-FAPI) demonstrated markedly higher uptake in diffuse GC and improved detection of both nodal and peritoneal metastases, frequently outperforming FDG in terms of lesion conspicuity and staging accuracy [35]. Although data remain preliminary, the rapid proliferation of FAPI-based studies suggests that FDG could potentially be partially supplanted by tumor stroma–targeted radiopharmaceuticals in this setting, especially for biologically aggressive and FDG-poor GC phenotypes [35].

### 4.4. MRI: The Most Promising Modality for Future Staging Algorithms

Among all contemporary imaging tools, MRI (particularly when incorporating diffusion-weighted imaging (DWI), intravoxel incoherent motion (IVIM), dynamic contrast enhancement (DCE), and whole-body DWI) has demonstrated significant potential to address the limitations of CT, EUS, and PET/CT [46,49]. Several consistent findings emerge. Beyond standard T1- and T2-weighted contrast, a key strength of MRI appears to be its multiparametric capability. Specifically, DWI provides a measure of microstructural restriction of water diffusion, reflected by the Apparent Diffusion Coefficient (ADC) map. This feature may be particularly relevant because low ADC values are inversely related to tumor cellularity and are sensitive to the desmoplastic reaction typical of diffuse or scirrhous gastric cancer (information that is not captured by conventional anatomical imaging (CT or EUS) [26].

#### 4.4.1. Superior Soft-Tissue Characterization

MRI facilitates a more detailed visualization of gastric wall layers and perigastric structures, improving assessment of depth of invasion in cases where CT is inconclusive [27].

This advantage is especially pronounced in scirrhous and poorly cohesive tumors, thickened yet poorly enhancing lesions, and cases with equivocal T2 vs. T3 transitions. Multiple prospective studies reported higher accuracy for T staging compared with CT in selected patients [26,27,46].

#### 4.4.2. Quantitative Biomarkers: ADC, DWI, and IVIM

Quantitative imaging signatures derived from ADC maps and advanced diffusion models consistently correlated with tumor grade, proliferative markers such as Ki-67, the degree of stromal desmoplasia, and molecular features including HER2 expression [98].

These metrics offer a non-invasive window into tumor biology, supporting the development of predictive models for aggressiveness, treatment response, and prognosis. Their reproducibility across institutions remains under investigation, yet radiomics-based and advanced multiparametric studies have shown encouraging external validity [57,99].

#### 4.4.3. MRI for Nodal Staging

Nodal characterization remains a major weakness across all modalities due to the poor specificity of size-based criteria. MRI, however, demonstrated higher sensitivity for metastatic nodes when incorporating signal-intensity features, ADC thresholds, and radiomics descriptors [100]. Although studies remain limited in size, results suggest a potential advantage over CT in biologically complex tumors [101].

#### 4.4.4. MRI for Peritoneal Metastases

The most striking contrast between MRI and CT emerges in the detection of peritoneal carcinomatosis. Several studies (including whole-body DWI and PET/MRI protocols) reported near-complete accuracy in identifying peritoneal implants and small-volume peritoneal disease, markedly outperforming CT and FDG PET/CT [67,102,103]. This finding is clinically relevant, given that peritoneal spread represents one of the leading causes of treatment failure and postoperative recurrence in gastric cancer.

#### 4.4.5. MRI After Neoadjuvant Therapy

Post-treatment evaluation is a rapidly expanding field. Advanced multiparametric MRI strategies (combining DWI, DCE, and high-resolution T2-weighted imaging, often with radiomics or histogram analysis) have demonstrated improved ability to differentiate viable tumors from treatment-induced fibrosis [99]. This suggests a potential role in selecting candidates for organ-preserving approaches or tailoring adjuvant therapy, thereby refining risk–benefit stratification in locally advanced gastric cancer [57].

#### 4.4.6. Radiomics and Artificial Intelligence: Toward Precision Imaging in GC

The integration of radiomics and machine-learning approaches across CT, MRI, and PET datasets has expanded rapidly over the last decade [104,105,106]. Radiomics captures subtle grayscale, textural, and shape features imperceptible to the human eye [107].

Several studies have indicated that radiomic signatures may outperform conventional imaging for predicting lymph node metastases [32,108] and correlate with tumor grade and molecular phenotype [36,109]. Additionally, these models show promise in stratifying the risk for peritoneal carcinomatosis [33,110] and predicting response to chemotherapy or chemoradiation [108].

Models built from MRI-derived features, particularly ADC heterogeneity metrics and diffusion-based texture indices, achieved the highest reproducibility and biological relevance, often outperforming size-based criteria and simple enhancement patterns [26,49]. Early deep learning models also showed promise for multi-class T staging, HER2 status prediction, and extranodal disease assessment, though their clinical applicability remains limited by small, often single-center datasets and lack of robust external validation [111,112]. A critical challenge in this field is ensuring the generalizability of these models across diverse institutions and patient populations, mitigating the risk of dataset bias that can undermine real-world predictive performance [105].

As quantitative imaging evolves, radiomics shows promise in complementing (not replacing) expert visual interpretation, helping refine staging accuracy and individualize therapeutic pathways [28].

#### 4.4.7. Current Challenges and Pitfalls in AI-Driven Staging

While AI and radiomics offer transformative potential, several pitfalls hinder their clinical translation. A primary concern is the lack of external validation; many models perform exceptionally well on internal datasets but fail when applied to diverse patient populations due to domain shift [113]. Furthermore, the ‘black box’ nature of deep learning often lacks interpretability, a critical factor for clinical trust. Adherence to standardized reporting guidelines, such as TRIPOD-AI (Transparent Reporting of a multivariable prediction model for Individual Prognosis or Diagnosis) [114] and CLAIM (Checklist for Artificial Intelligence in Medical Imaging) [115], remains suboptimal in many early studies. Addressing these issues through multicenter collaborations and open-source data harmonization is essential to bridge the ‘AI chasm’ between research and routine oncology practice.

#### 4.4.8. Influence of Histotype on Imaging Accuracy

One of the strongest themes across modalities is the profound impact of histopathology on diagnostic accuracy [116,117].

Diffuse-type GC presents distinct morphological challenges, characterized by submucosal infiltration without discrete mass formation, low contrast enhancement on CT, and poor FDG uptake on PET/CT. Additionally, reduced layer stratification on EUS is common due to early transmural spread and the desmoplastic reaction [34,118].

These biological and morphological traits explain why diffuse tumors are consistently understaged across CT, PET/CT, and EUS, with frequent underestimation of T category, nodal involvement, and peritoneal dissemination [110]. MRI (particularly through diffusion-derived parameters, whole-tumor ADC analysis, and multiparametric protocols) appeared to be more resilient to these limitations, showing potentially higher sensitivity for infiltrative wall thickening and occult peritoneal disease in diffuse and poorly cohesive histotypes [57,98]. Staging laparoscopy studies further confirm that diffuse and poorly cohesive Lauren subtypes carry a higher burden of radiologically occult peritoneal metastases, reinforcing the need for tailored staging strategies in this group [119,120].

This underscores the need for histotype-informed imaging pathways, rather than uniform staging algorithms for all gastric cancers, integrating CT, MRI, PET/CT, and staging laparoscopy according to the underlying biological profile and risk of occult metastatic spread [121].

#### 4.4.9. Integrated Multimodality Approach: Redefining the Staging Workflow

Based on the aggregated evidence, a modern staging algorithm for GC should be adaptive. CT serves as the universal first-line modality for global assessment, while EUS is reserved primarily for early lesions or when CT suggests superficial invasion. MRI should be specifically considered in diffuse-type or poorly cohesive tumors, for ambiguous CT findings, suspected peritoneal disease, and for pre- and post-neoadjuvant evaluation. Regarding nuclear medicine, PET/CT remains useful for distant metastases in intestinal-type tumors, whereas FAPI PET is increasingly positioned as the optimal tracer for diffuse and scirrhous tumors.

**Figure 2:**
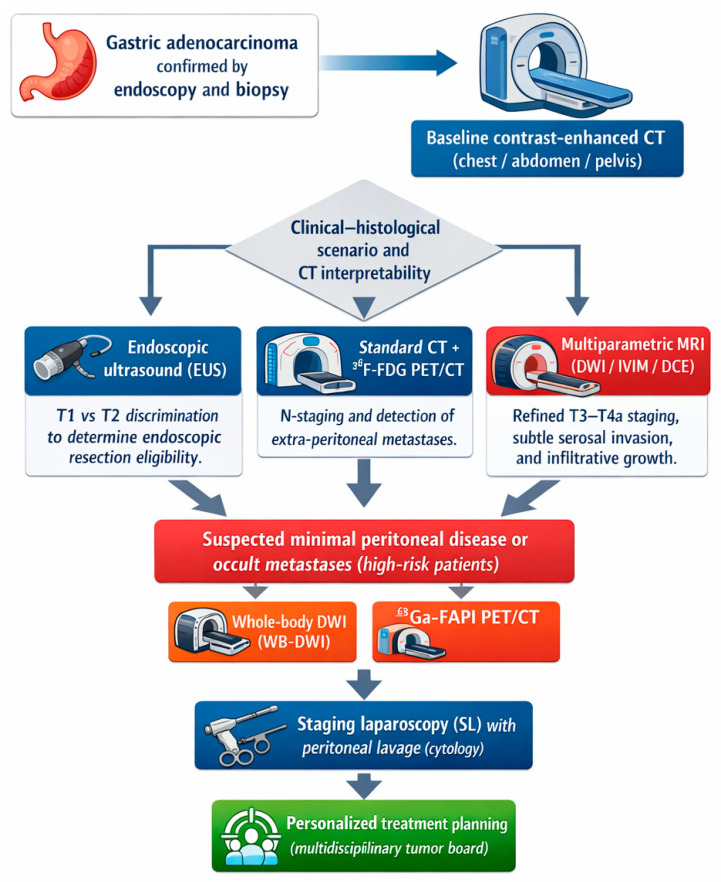
Proposed Histotype-Adapted Diagnostic Algorithm for Gastric Cancer Staging. This figure illustrates a tailored staging workflow. While CT remains the baseline modality for all patients, the integration of EUS is prioritized for early lesions, whereas multiparametric MRI (specifically DWI) and advanced PET tracers (FAPI) are positioned as crucial problem-solving tools for diffuse histotypes and the detection of occult peritoneal spread.

Such a multimodal strategy aligns with precision oncology principles, maximizing staging accuracy and avoiding understaging that may compromise oncological outcomes (Figure 2).

#### 4.4.10. Key Gaps and Future Directions

Despite the significant progress documented, several unmet needs persist, including the lack of large prospective multicenter trials validating MRI and radiomics-based models and the limited availability of WB-DWI/MRI in routine clinical practice. Furthermore, there is insufficient standardization of acquisition and reconstruction parameters across platforms, scarce data on post-neoadjuvant MRI response assessment, and a need for broader evaluation of emerging PET tracers such as FAPI. Finally, specific histotype-driven imaging guidelines are still lacking.

Beyond technical validation, cost-effectiveness and resource allocation remain pivotal for clinical implementation. While CT is universally accessible and relatively inexpensive, multiparametric MRI and FAPI-PET involve higher costs and require specialized expertise. However, in high-risk scenarios (such as diffuse-type GC), the potential to avoid unnecessary surgical explorations or inadequate neoadjuvant treatment through more accurate staging may justify the higher initial imaging expenditure. Furthermore, while current international guidelines (e.g., NCCN, ESMO, AJCC) primarily emphasize the CT-EUS-PET triad, the evidence synthesized here supports a transition toward more personalized, histotype-driven staging pathways, which could refine future guideline updates.

Future research should prioritize harmonization of imaging protocols, integration of AI-assisted decision tools, and structured evaluation of multi-modality algorithms in diverse patient populations.

#### 4.4.11. Summary of Implications

This review indicates that contemporary imaging of gastric cancer is evolving beyond the traditional CT–EUS–PET triad, with MRI (and particularly diffusion and perfusion techniques) emerging as the most promising tool for accurate, biologically informed staging. As evidence continues to expand, MRI may assume a more central role within international guidelines, particularly for diffuse histotypes and peritoneal disease, where it consistently outperforms other modalities.

The integration of advanced PET tracers and quantitative MRI biomarkers reinforces a paradigm shift toward precision imaging, where staging reflects not only the anatomical extent but also the biological behavior of the tumor.

Finally, it is important to acknowledge that while advanced modalities such as MRI and AI-based radiomics show promising results, a significant portion of the current evidence stems from retrospective, single-center studies. Consequently, these findings should be interpreted as strong indicators of potential rather than definitive proof of clinical superiority. Further standardized, prospective trials are mandatory to establish their definitive place in international staging guidelines.

## 5. Conclusions

Accurate preoperative staging in gastric cancer remains a critical determinant of therapeutic decision-making and prognostic assessment. The evidence synthesized in this expanded and comprehensive narrative review (integrating updated literature (2015–2024), quantitative imaging approaches, and histotype-specific considerations) suggests that no single modality provides uniformly reliable staging across the biological diversity of gastric cancer.

CT remains indispensable as first-line imaging, but its limitations can become evident in diffuse and poorly cohesive tumors, in nodal characterization, and in the detection of low-volume peritoneal disease. EUS continues to serve as the most accurate technique for early T staging, although its performance declines substantially in advanced disease and after neoadjuvant therapy.

In contrast, MRI is emerging as a modality offering potentially substantial incremental value, particularly through diffusion- and perfusion-based techniques, which provide superior soft-tissue contrast and biologically meaningful quantitative biomarkers. MRI indicates clear advantages in distinguishing T2 from T3 disease, improving nodal assessment beyond size-based criteria, and markedly outperforming CT and FDG PET/CT in the detection of peritoneal carcinomatosis. These strengths make MRI an essential adjunct in challenging histotypes, especially the diffuse subtype, where traditional modalities consistently underestimate disease extent.

Parallel advancements in radiomics and artificial intelligence further expand the potential of cross-sectional imaging, enabling the extraction of subtle features linked to tumor heterogeneity, molecular phenotype, and treatment response. Although external validation remains limited, these quantitative approaches represent a decisive step toward precision imaging and individualized care pathways.

Overall, the current evidence supports a tailored, multimodality staging algorithm in which CT provides global assessment, EUS is selectively applied for superficial disease, MRI is incorporated for biologically or anatomically complex presentations, and PET (particularly with emerging tracers such as FAPI) enhances detection of distant or peritoneal spread. Future research should prioritize prospective multicenter validation, harmonization of acquisition protocols, and integration of AI-derived biomarkers into clinical workflows.

Collectively, these developments point toward a transition toward a more biologically informed imaging paradigm, in which the choice and sequencing of modalities are adapted to the intrinsic behavior of each tumor. Such an approach is essential for improving staging accuracy, optimizing therapeutic selection, and ultimately enhancing clinical outcomes for patients with gastric cancer.

## Figures and Tables

**Table 1:** Key representative studies included in the final evidence set (*n* = 410).

Author, Year	Modality/Theme	Key Contribution	Relevance to Gastric Cancer Staging	Level of Evidence (OCEBM)
Arslan et al., 2017 [26]	MRI (Advanced sequences, Staging Review)	DWI/perfusion MRI outperforms CT in T/N staging; Defines best MRI sequences for standardization.	Establishes MRI as emerging standard; provides standardization guidance.	Moderate
Joo et al., 2015 [27]	PET/MRI vs. MDCT	PET/MRI improves T staging and resectability	Supports hybrid imaging	High
Zhang et al., 2025 [28]	CT texture analysis	Texture features correlate with T stage	Early radiomics evidence in CT	Moderate
Küpeli et al., 2019 [29]	Dual-energy CT	DECT improves depiction of serosal invasion	Improves T4 differentiation	High
Song et al., 2015 [30]	PET/CT & CECT	PET improves M staging; CT is superior for T	Establishes complementarity	Low
Chen et al., 2023 [31]	PET/MR FAPI vs. FDG	FAPI has higher lesion contrast	Beneficial in scirrhous/diffuse types	High
Jiang et al., 2025 [32]	Radiomics (LN metastasis)	PET radiomics improves LN staging	Improving LN staging	Moderate
Shi et al., 2024 [33]	CT + PET radiomics	Predicts lymphovascular invasion	Pre-operative biologic risk profiling	High
Hayes et al., 2017 [2]	Staging principles	Synthesizes role of CT, EUS, and PET	Benchmark recommendations	Moderate
Leeman et al., 2017 [34]	Peritoneal metastasis detection	Laparoscopy can be superior to imaging	Essential comparison for PM detection	High
Kiran et al., 2024 [35]	PET/FAPI vs. FDG	Confirms FAPI superiority	Robust pooled evidence	High
Guan et al., 2022 [36]	HER2 expression and radiomics	Deep learning in high HER2 expression	Enhance the preoperative staging using AI	Moderate
Song et al., 2015 [37]	PET volumetric parameters	MTV/TLG prognostic for survival	PET-derived prognostic modeling	Moderate
Shen et al., 2023 [22]	Delta radiomics for advanced gastric cancer	Radiomics improves PM prediction	Quantitative enhancement	Moderate
Mikami et al., 2017 [10]	Bone lesions detection	Marrow uptake correlates with recurrence	Systemic disease imaging	Moderate

**Table 2:** Diagnostic accuracy of imaging modalities for gastric cancer staging.

Modality	Key Evidence Sources	T-Stage Accuracy	N-Stage Accuracy	M-Stage Accuracy/Specific Role	Notes/Strengths/Limitations
Contrast-Enhanced CT (CECT)	Hayes 2017 [2]; Yu 2015 [39]; Li 2017 [52]; Saito 2015 [53]	65–80% for T3–T4; limited for T1–T2	50–70%	Moderate for distant metastasis, good for liver/lung	First-line modality; limited soft-tissue contrast; difficulty differentiating T2–T3 and assessing serosa (T4a).
Dual-Energy CT (DECT)	Küpeli 2019 [29]	Up to 85% for serosal invasion	60–75%	Comparable to CT; enhanced iodine mapping for tumor conspicuity	Better depiction of mural infiltration; emerging modality.
EUS (Endoscopic Ultrasound)	Sacerdotianu 2022 [40]; Li 2017 [14]; Ungureanu 2021 [54]; de Nucci 2023 [41]	75–90% overall; best for T1–T2	50–65%	Not routinely used for M staging	Superior performance for depth of invasion in early GC; operator-dependent; reduced accuracy after neoadjuvant therapy.
Double Contrast-Enhanced US	Wang 2021 [55]	82–90%	65–75%	Limited	Useful when EUS is unavailable; performance similar to EUS for T staging.
MRI (conventional + DWI)	Arslan 2017 [26]; Joo 2015 [47]; Giganti 2016 [56]; Méndez 2024 [57]	80–95% for T3–T4; best for serosal invasion (T4a)	65–85% (DWI improves N staging)	Moderately useful for PM; better than CT for occult metastasis	Superior soft-tissue contrast; robust for distinguishing T2/T3 and T3/T4.
Whole-Body DWI/WB-MRI	De Vuysere 2021 [46]	—	—	90–94% for metastatic disease; high sensitivity for PM	Superior non-invasive alternative to staging laparoscopy for peritoneal metastases.
IVIM-DWI/advanced MRI models	Hong 2024 [58]; Li 2025 [59]; Zeng 2021 [60]	85–92% (improves differentiation of T2 vs. T3)	75–90%	Helpful for micro-metastatic spread	Quantitative microvascular/tissue diffusion biomarkers.
DCE-MRI (perfusion MRI)	Giganti 2016 [56]; Tang 2020 [61]; Zhu 2021 [62]	80–93%	—	Limited	Perfusion metrics correlate with aggressiveness and extramural venous invasion.
FDG-PET/CT	Findlay 2019 [63]; Altini 2015 [64]; Wang 2016 [65]	Poor for T staging	55–65%	High specificity for M staging; 70–90%	Essential for distant metastasis; limited sensitivity for signet-ring/diffuse types.
FDG-PET/MRI	Lee 2016 [66]; Yoon 2021 [67]	70–80%	70–80%	Improved M staging vs. CT	Benefits from MRI’s soft-tissue contrast; useful for evaluating resectability.
FAPI-PET (68Ga-FAPI and FAPI-74)	Huang 2023 [68]; Du 2023 [69]; Ruan 2023 [70]; Kuten 2022 [71]	Superior lesion-to-background ratios; not used for T staging	Potentially high	Superior non-term imaging test for peritoneal metastasis; detects occult lesions missed by CT/MRI/FDG	Excellent sensitivity for scirrhous and mucinous GC; rapidly emerging as a transformative modality.
CT Radiomics	Zhang 2022 [72]; Fan 2022 [73]; Chen 2024 [74]	Improves T-stage discrimination (AUC 0.80–0.92)	AUC 0.78–0.90 for LN metastasis	Predicts PM when combined with clinical variables	Extracts intratumoral heterogeneity not visible on CT.
MRI Radiomics	Chen 2019 [75]; Li 2025 [59]	T-stage AUC up to 0.94	N-stage AUC 0.88–0.93	Predicts EVMI and prognosis	Higher dimensionality than CT; more stable features.
PET Radiomics/PET–CT Radiomics	Xue 2022 [76]	—	AUC 0.82–0.90	Improves detection of PM and prognosis	Quantitative metabolic features outperform simple SUV metrics.
AI/Deep Learning (CT, MRI, PET)	Li 2022 [77]; Garbarino 2024 [78]	AUC 0.85–0.95 for T staging	AUC 0.88–0.94 for N staging	Predicts PM, TRG response, and survival	Next-generation predictive tools; highest AUC values are typically reported for binary classification tasks (e.g., T4 vs. non-T4, N+ vs. N−), but models require multicenter validation.

Notes: Accuracy metrics (percentages) represent overall diagnostic accuracy reported in key studies, while AUC values are included for quantitative imaging models (Radiomics/AI) to denote discriminative performance.

**Table 3:** Summary of imaging modalities strengths and weaknesses in gastric cancer staging.

Modality	Key Strengths	Main Limitations/Notes
CT	Good first-line modality for global staging	Limited accuracy in diffuse or poorly cohesive subtypes, nodal staging, and detection of small distant metastases
EUS	Highest accuracy for early disease (T1–T2) and for selecting candidates for endoscopic resection	Reduced performance in advanced tumors, cardia/pylorus lesions, and after neoadjuvant therapy
FDG PET/CT	High specificity for distant metastases and useful to confirm unexpected extra-peritoneal disease	Poor sensitivity for diffuse histotypes and peritoneal metastasis, with low uptake in signet-ring/diffuse gastric cancer
MRI	Superior soft-tissue contrast and excellent performance for peritoneal metastasis and nodal staging, providing quantitative biomarkers (e.g., ADC, IVIM, DCE) predictive of tumor biology and a robust platform for radiomics and deep learning models	Still less available than CT, and protocol/post-processing standardization is ongoing
FAPI PET	Higher uptake than FDG in low-uptake and stroma-rich tumors and an emerging role in staging, particularly for diffuse gastric cancer	Evidence is still preliminary, with limited availability and lack of long-term outcome data
Radiomics/AI	Promising tools for robust quantitative staging and risk stratification, especially when using multiparametric MRI and CT datasets	Clinical implementation is limited by small, often single-centre cohorts, methodological heterogeneity, and scarce external validation

## Data Availability

Data sharing is not applicable to this article as no new data were created or analyzed during this study.
